# Safety and Efficacy of Human Wharton's Jelly-Derived Mesenchymal Stem Cells Therapy for Retinal Degeneration

**DOI:** 10.1371/journal.pone.0128973

**Published:** 2015-06-24

**Authors:** S. N. Leow, Chi D. Luu, M. H. Hairul Nizam, P. L. Mok, R. Ruhaslizan, H. S. Wong, Wan Haslina Wan Abdul Halim, M. H. Ng, B. H. I. Ruszymah, S. R. Chowdhury, M. L. C. Bastion, K. Y. Then

**Affiliations:** 1 Department of Ophthalmology, Universiti Kebangsaan Malaysia Medical Centre, Kuala Lumpur, Malaysia; 2 Centre for Eye Research Australia, University of Melbourne, Royal Victorian Eye & Ear Hospital, Melbourne, Australia; 3 Department of Obstetrics & Gynaecology, Faculty of Medicine and Health Sciences, Universiti Putra Malaysia, Selangor Darul Ehsan, Malaysia; 4 Genetics & Regenerative Medicine Research Centre, Faculty of Medicine and Health Sciences, Universiti Putra Malaysia, Selangor Darul Ehsan, Malaysia; 5 Tissue Engineering Centre, Universiti Kebangsaan Malaysia Medical Centre, Kuala Lumpur, Malaysia; Wake Forest Institute for Regenerative Medicine, UNITED STATES

## Abstract

**Purpose:**

To investigate the safety and efficacy of subretinal injection of human Wharton’s Jelly-derived mesenchymal stem cells (hWJ-MSCs) on retinal structure and function in Royal College of Surgeons (RCS) rats.

**Methods:**

RCS rats were divided into 2 groups: hWJ-MSCs treated group (n = 8) and placebo control group (n = 8). In the treatment group, hWJ-MSCs from healthy donors were injected into the subretinal space in one eye of each rat at day 21. Control group received saline injection of the same volume. Additional 3 animals were injected with nanogold-labelled stem cells for *in vivo* tracking of cells localisation using a micro-computed tomography (microCT). Retinal function was assessed by electroretinography (ERG) 3 days before the injection and repeated at days 15, 30 and 70 after the injection. Eyes were collected at day 70 for histology, cellular and molecular studies.

**Results:**

No retinal tumor formation was detected by histology during the study period. MicroCT scans showed that hWJ-MSCs stayed localised in the eye with no systemic migration. Transmission electron microscopy showed that nanogold-labelled cells were located within the subretinal space. Histology showed preservation of the outer nuclear layer (ONL) in the treated group but not in the control group. However, there were no significant differences in the ERG responses between the groups. Confocal microscopy showed evidence of hWJ-MSCs expressing markers for photoreceptor, Müller cells and bipolar cells.

**Conclusions:**

Subretinal injection of hWJ-MSCs delay the loss of the ONL in RCS rats. hWJ-MSCs appears to be safe and has potential to differentiate into retinal-like cells. The potential of this cell-based therapy for the treatment of retinal dystrophies warrants further studies.

## Introduction

Inherited retinal degenerative diseases such as retinitis pigmentosa (RP) are the major cause of irreversible blindness worldwide. Currently, there is no effective treatment either for preventing or slowing the progression of the disease. Genetic therapy had been challenging as there is a wide range of genetic mutations involved and targeting every individual mutations is technically difficult. Cell based therapy seems to be a promising strategy in RP as it has the potential to regenerate new photoreceptors or retinal pigment epithelial (RPE) cells. Several types of stem cells had been investigated. However, in vivo studies showed that cells derived from human umbilical cord tissue appears to be the most effective in rescuing photoreceptors and retinal function [1].

Fetal stem cells are of different entities and can be obtained from two distinct sources, namely the fetus proper (fetal bone marrow[2], lung[3],spleen, liver[4]and peripheral blood[5]) and umbilical cord tissue (e.g umbilical cord blood[6], Wharton’s jelly, amniotic fluid[7], placenta[8] and amnion[9]). Umbilical cord tissue itself harbours different stem cell population in its many compartments namely amnion, subamnion, Wharton’s jelly, perivascular, adventitia, endothelium and umbilical cord blood and the differences in stemness characteristics have been reported[10,11].

Stem cells derived directly from uncontaminated Wharton’s jelly are less heterogenous and possess unique beneficial properties over other mesenchymal stem cells[11–15]. Human Wharton’s Jelly-derived Mesenchymal Stem Cells (hWJ-MSCs) in its pure form have many advantages over other type of stem cells including higher proliferation rates, stemness characteristics that lasts several passages in vitro, wide multipotency, hypoimmunigenicity and anticancer properties[12]. hWJ-MSCs evoked minimal immune reactivity with low expression of MHC I molecules and no expression of MCH II molecules; rendering them a good source of allogeneic cell transplantation[14,16]. hWJ-MSCs has greater differentiation potential [10,12,17] than other tissues in umbilical cord. The potential of hWJ-MSCs to differentiate into neurons[17–20] especially retinal progenitor cells[21] is a promising feature in cell therapy for conditions such as retinal degeneration. Apart from that, hWJ-MSCs can also synthesize and secrete trophic factors or cytokines and to support the expansion and function of other neural cells[17,18,20,22]. Trophic factors secreted by hWJ-MSCs showed a better neural differentiation and neural cell migration when compared with trophic factors by bone marrow-derived mesenchymal stem cells [23].

hWJ-MSCs has been studied widely in many conditions such as ischemic stroke[24], spinal cord injury[25], Parkinson disease[26], cardiovascular disease[27,28], cartilage disease[29], liver injury[30], skin healing[31]. However, the application of hWJ-MSCs in its pure form for treating retinal degenerative diseases have not been studied previously. Thus, the purpose of this study was to investigate the safety and efficacy of subretinal injection of hWJ-MSCs on preservation of the outer retinal structure and function in a rat model of retinal degeneration.

## Methods

The study was approved by the Universiti Kebangsaan Malaysia Animal Ethnics Committee (UKMAEC) (Approval number: PP/OPHTAL/2011/HASLINA) and all experiments were conducted in accordance with the guidelines drawn by UKMAEC and also conformed to the ARVO Statement for the Use of Animals in Ophthalmic and Vision Research.

### Animals

Pigmented dystrophic Royal College of Surgeons (RCS) rats were obtained from the National BioResource Project (NBRP) for the Rat in Japan (NBRP Rat no: 0011, Kyoto, Japan). RCS rats have a causative gene *rdy* causing a deletion in the Mertk gene encoding the tyrosine kinases receptor. This results in retinal abnormality starting at 3 weeks of age where RPE fails to phagocytose shed photoreceptor cells and subsequently leads to apoptosis of photoreceptors; mimicking human retinal degeneration[32,33]. Animals were housed in animal laboratory in Universiti Kebangsaan Malaysia with a 12-hour light/dark cycle in individually ventilated cages. The day of injection was considered as P0. All interventions were performed under full anaesthesia with intramuscular injection of ketamine (80 mg/kg) and xylazine (7.5 mg/kg) [34].

### Cell preparation

In this study, we use a more defined mesenchymal stem cells that is derived from human Wharton’s Jelly rather than the whole umbilical cord tissue. The cell source used is also cultured using animal-free culture media that is important in developing a cell based therapy for human use.

Human Wharton’s Jelly mesenchymal stem cells from a single donor were obtained from a local stem cell bank (CryoCord Malaysia). Human umbilical cord was collected from the donor at full-term gestation with the mother’s consent for research purpose. All the cell preparation were done in a cGMP accredited laboratory. Umbilical cord blood was drained before cord tissue pieces were cut open lengthwise. The umbilical cord Wharton’s Jelly will be shredded and then enzymatically digested using collagenase (Worthington Biochem, USA) for 1–2 hours at 37°C to facilitate detachment and loosening of the Wharton’s jelly into culture medium. The mesenchymal cells were isolated from Wharton’s jelly by passing the tissue through a syringe and needle. They were then cultured in Dulbecco’s modified Eagle’s medium (DMEM)-low glucose (Gibco,USA)+10% human serum+100U/ml penicillin and 100μg/ml streptomycin and 0.25μg/ml amphotericin (Gibco,USA). The culture-expanded cells were also cryopreserved using standard cryopreservation protocol until being used in the following research experiment.

Average cell viability upon thawing was 92.1%. The cells were then characterized with flow cytometric analysis, gene expression with real-time polymerase chain reaction (PCR) and multilineage differentiation tests according to International Society for Cellular Therapy (ISCT) criteria for mesenchymal stem cells[35]. Immunophenotyping with flow cytometric analysis showed positive surface markers expression of CD90, CD105 and CD73. The cells were negative for hematopoietic markers of CD34 and CD45 as well as low expression for HLA-DR (S1 Fig). Real-time PCR showed expression of stemness genes relative to glyceraldehyde-3-phosphate dehydrogenase (GAPDH) as a reference gene; namely Sox-2, Nanog 3, Nestin, BST-1, OCT-4, FZD-9, FGF-4, Rex-1 and ABCG2 (S1 Table). Differentiation tests showed the ability of hWJ-MSCs to differentiate into all three lineages: adipogenic, osteogenic and chondrogenic by staining positively for Oil Red O, Alizarin Red S and Alcian Blue respectively (S2 Fig). Apart from that, hWJ-MSCs were also tested for differentiation into neurons. They were incubated in α-MEM containing 1 mM b-mercaptoethanol (BME) without serum for 24 h. The culture medium was then replaced with differentiation medium consisting of α-MEM containing 10% FBS and 35 ng/ml all-trans-retinoic acid (ATRA). Three days later, cells were finally transferred to α-MEM containing 10% FBS and trophic factors (5 mM forskolin, 10 ng/ml recombinant human basic fibroblast growth factor (bFGF), 5 ng/ml platelet- derived growth factor-AA (PDGF-AA) and 200 ng/ml heregulin-b1-EGF-domain and cultured for an additional 8 days before they were ready for in vitro evaluation. Both the induced cells and non-induced cells were then counterstained with glial fibrillary acidic protein (GFAP) and nestin and viewed with confocal microscopy. hWJ-MSCs stained positive for both GFAP and nestin which are markers for neurons (S3 Fig). Cells at passages 4 to 5 were used throughout this experiment.

### Preparation of gold nanoparticles-loaded hWJ-MSCs

The quality of the gold nanoparticles used in this study was assessed by determining the optimal absorption wavelength of the gold nanoparticles in different culture medium with a spectrophotometer. The best absorption wavelength was to enable reliable results when measuring the concentration of gold nanoparticles. Following 24 hours incubation of gold nanoparticles on hWJ-MSCs, the supernatants were harvested and the amount of gold nanoparticles in it were diluted in water before being measured at optimal absorption wavelength. This is to measure the gold nanoparticles released by the cells (exocytosis). Gold nanoparticle (BBI Internationals) solutions were centrifuged and the pellet resuspended in phenol red free medium at a concentration of 1x10^9^ nanoparticles/ml. The hWJ-MSCs culture media was then removed from the flasks and 200 μl/cm^2^ of nanogold particle medium (1x10^9^ particles/ml) was then added to the cell culture flask and left to be incubated for a day. The supernatant containing nanogold particles were then removed after 24 hours. The nanogold-loaded hWJ-MSCs were washed with phosphate-buffered saline.

Cell viability for hWJ-MSCs after gold nanoparticles loading was evaluated with LIVE/DEAD staining. Stock solution of calcein AM and ethidium homodimer-III were diluted with phosphate-buffered saline according to the manufacturer’s (Biotium.Inc, USA) recommendations to a concentration of 2μM and 4μM respectively. hWJ-MSCs growth medium was aspirated from the wells and the cells were washed two to three times with phosphate-buffered saline. Incubation of cells in the LIVE/DEAD stain was performed for 45 min at room temperature. They were then washed two to three times with phosphate-buffered saline. The stained cells were then viewed with fluorescence microscopy (Leica DMI2000B microscope equipped with a Leica DF290 camera) under 10x magnification. Controls are cells not loaded with gold nanoparticles. Viability of cells was then evaluated at days 1, 5 and 10 which correlated to the number of days after the gold nanoparticles loading.

Cell proliferation assay was done using CellTiter-Glo Luminescent Cell Assay (Promega, USA). Briefly, CellTiter-Glo reagent was prepared according to the manufacturer’s recommendations and added to cells at a concentration of 100 μl/mL of medium. The samples were incubated according to the culture protocol. The plate and its contents were left at room temperature for 30 min. An equivalent volume of CellTiter-Glo reagent to the volume of cell culture medium in each well was added and mixed for few min on an orbital shaker to induce cell lysis. The plate was then left to incubate at room temperature for 10 min to stabilise the luminescent signal. The luminescence was then recorded with GloMax luminometer. Cells not incubated with gold nanoparticles acted as controls. A blank sample with no cells was used and deducted from all measurements. Cytotoxicity was evaluated at day 1, day 5 and day 10. Triplicate samples were used and the assay was repeated twice.

### Electroretinography

Electroretinography was performed 3 days before the injection (day -3) and repeated at days 15, 30 and 70 post injection. We used the day of injection as reference day 0. Animals were dark adapted overnight for at least 12 hours before being anesthetized with intramuscular injection of ketamine (80 mg/kg) and xylazine (7.5 mg/kg). Topical anesthesia of tropicamide 0.5% and phenylephrine 2.5% were used to dilate the pupils. Body temperature was kept at 36°C with a warm blanket.

ERGs were recorded with a gold wire loop placed on cornea and good contact between cornea and electrode was assured with a drop of saline. The reference gold-cup electrode was placed in the mouth and the ground needle electrode was inserted to the tail. Preparation for ERG recordings were conducted under dim red light.

All recordings were performed using an Espion system (Espion e2, Diagnosys LLC; USA). An average of at least three consistent recordings was taken for each stimulus intensity. The stimulus intensity ranged from -4.0 to 1.0 log cd.s/m^2^. In addition, a double flash recording protocol presented at 1.0 cd.s/m^2^ with the inter-stimulus interval of 0.7s was performed to obtain an isolated cone response.

### Cell Injection

Cells were trypsinized, washed and suspended in balanced salt solution prior to injection. The viability of cells were tested *in vitro* with trypan blue staining after passing through a 30G needle as cell lysis was a concern. At 30G, the cells showed a viability of 80–90%.

At the age of days 21–23, 8 RCS rats kept under xylazine-ketamine anaesthesia received a trans-scleral subretinal injection of a suspension of hWJ-MSCs 2 μl (1x10^5^ cells/μl) (S4 Fig). The procedure was visualized with an operating microscope through a dilated pupil. The site of injection was exposed using a traction suture on the conjunctiva (Figure A in S4 Fig). Conjunctival periotomy was done at the injection site superotemporally (Figure B in S4 Fig) and a subretinal injection of cells was done with a 30G Hamilton syringe (Figure C in S4 Fig) after a scleral tunnel was created using an insulin needle. The site of injection was marked with a 10/0 nylon suture (Figure D in S4 Fig). Immediately after the injection, the fundus was examined to check for complications and if present, the animal would be removed from the study.

All animals were given immunosuppression to reduce the risk of cell rejection. They were given daily dexamethasone injections intraperitoneally (1.6 mg/kg, from the day of surgery) for 2 weeks. Cyclosporine-A (Bedford Labs, Bedford, MA) was added to their drinking water (210 mg/l; resulting in blood concentration of 250–300 μg/l from a day prior to injection to the length of the entire experiment in order to reduce the risk of cell rejection. Eight RCS rats were injected with hWJ-MSCs and another eight with balanced salt solution (BSS). Three rats were injected with gold nanoparticles-loaded hWJ-MSCs.

### Micro-computed tomography

Biodistribution of the stem cells after transplantation are important aspects to look into in terms of safety profile cellular therapy. Apart from local distribution, we are also interested to know whether the stem cells will home in or migrate to other parts of the body and their final location. C.Villa had compared various modalities of *in vivo* tracking of stem cell by nanotechnologies ranging from magnetic resonance imaging, bioluminescence imaging to micro computed imaging and found microCT to be a good method for *in vivo* tracking in murine animals[36,37]. MicroCT uses the all-embracing universal application of x-ray imaging method which has high spatial resolution of distribution of nanoparticles labelled stem cells and reconstruction of 3D images. However, they used iron oxide nanoparticles in their study. Iron oxide had been shown to be taken up by stem cells and undergone lysosome degradation which releases free iron from the cytoplasm [38]which is toxic to the retina. Therefore, in this study, we choose gold nanoparticles which is inert and has good optical properties. Furthermore, gold had been used to track stem cell in renal study [39].

Micro-computed tomography were performed on 3 rats injected with gold nanoparticles-loaded hWJ-MSCs using Skyscan 1076 at days 1, 30 and 70 post injection. The rats were anaesthesized and strapped to the holder. The source spot size was 8.88um at 45kVp (with 0.5 mm Al filtering). We injected the rats with 2μl of gold loaded hWJ-MSCs at the gold nanoparticles concentration of 1x10^9^ particles/μl.

### Histology

The rats were sacrificed at day 70 post injection for enucleation. Animals were euthanized with sodium pentobarbital intravenously at a dose of 15mg/100g body weight and enucleation performed. The enucleated eyes were immersed in 4% paraformaldehyde for an hour before embedded in Optimal Cutting Temperature (OCT). The eyes were then cut in horizontal sections at 10μm thickness using cryostat and mount on glass slides. Hematoxylin and eosin staining were then performed. The outer nuclear layer (ONL) thickness at three different sites were measured using QCapture Pro Software and averaged.

As for the electron microscopy, the eye cups were immediately fixed with 2.5% glutaraldehyde solution (pH 7.4) in 0.1M phosphate buffer for an hour and then treated with 1% osmium tetroxide solution (OsO_4_) in 0.1M phosphate buffer for an hour. The eyes samples were then dehydrated with graded ethanol and treated with epoxy propane and embedded in 812 resin. The embedded retinas were sectioned serially for electron microscopy on an ultramicrotome (Leica EM UC6, using a diamond knife. Semi-thick sections (500nm) were stained with 1% Toluidine blue and viewed under microscope to confirm the site of interest. The ultra-thin (76nm) series of sections were collected on a slot grid, stained with uranyl acetate and lead citrate, and washed. The grids were viewed and serial sections were photographed under a Tecnai G2 Spirit Biotwin (FEI Company, USA). The area of the retina distant from the graft was used as a matching negative control for each eye.

### Immunohistology Staining

The frozen sections of tissues were used for immunohistological staining. The tissues were cut at 10μm thick and fixed in 4% paraformaldehyde. The slides were thawed at room temperature for 30 min and tissue sections rehydrated with PBS for 15 min. The section were permeabilized with PBS containing 0.2% Triton X-100 (PBST) for 20 min at room temperature. Non-specific binding sites were blocked with 0.2% bovine serum albumin (BSA) (Zenon Mouse IgG Labelling Kit) and 5% of heat inactivated normal goat serum for 30 minutes. The sections were then prepared with Zenon’s labelling complex according to the manufacturer’s recommendation. (Molecular Probes Invitrogen Detection Technologies, 2008) The tissues were then counterstained with primary mouse monoclonal antibody-STEM121 (StemCells Inc, USA) specific for human cytoplasmic marker to highlight the hWJ-MSCs, anti-rhodopsin antibody for rod photoreceptors, anti-protein kinase C (PKC-α) antibody for bipolar cells, anti-glial fibrillary acidic protein (GFAP) antibody for Muller glial cells, anti-cone arrestin antibody for cone photoreceptors, anti-recoverin antibody for cone bipolar, anti-microphthalmia-inducing transcription factor (MITF) antibody for RPE specific markers and anti-ß-tubulin III antibody for immature neurons (all from Santa Cruz Biotech Inc, Europe). Except for STEM 121, all other antibodies stained both human and rat proteins. The confocal images were viewed with Leica Application Suite Advanced Fluorescence (LAS-AF) system.

### Statistical Analysis

Data were presented as mean ± standard error of the mean. Mann-Whitney U test was used for comparison of cell proliferation and ERG responses. Student’s t-test was used for comparison of ONL thickness. Significance level was designated as p ≤ 0.05.

## Results

### Outer Nuclear Layer (ONL) Thickness

**Fig 1:**
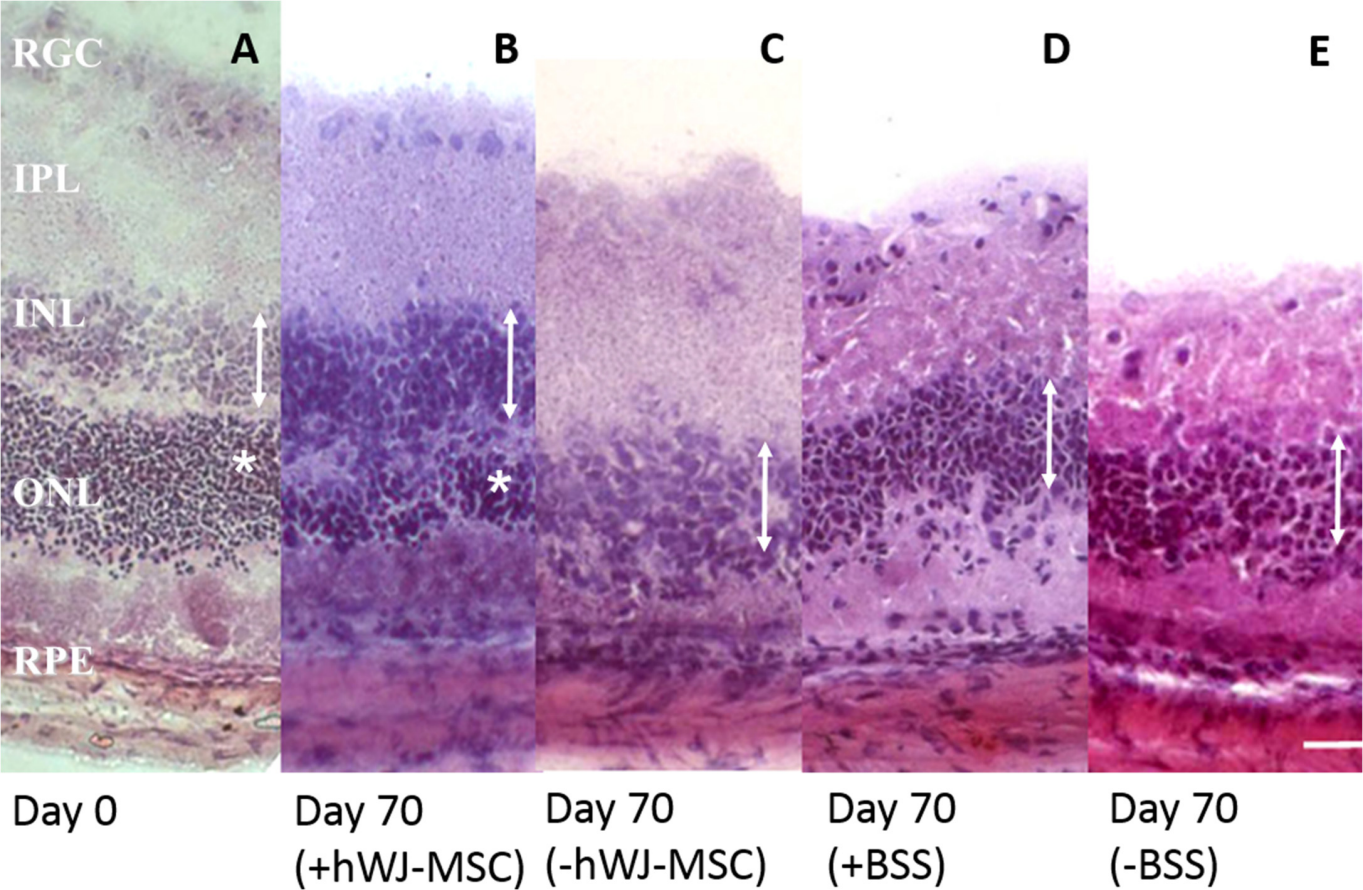
Histology of experimental groups. A representative histology at day 0 (A) and day 70 (B-E) of different experimental groups. At day 70, the ONL were clearly detectable in eyes treated with hWJ-MSCs (B) compared to a thin layer or almost absent ONL in contralateral non-injected eye (C). The ONL was undetectable in the BBS injected eye (D) or the follow control eye (E). RGC: retinal ganglion cell, IPL: inner plexiform layer, INL: inner nuclear layer (indicated by double-headed arrows), ONL: outer nuclear layer (indicated by asterisks), RPE: retinal pigment epithelium. Scale bar represents 20μm.

**Fig 2:**
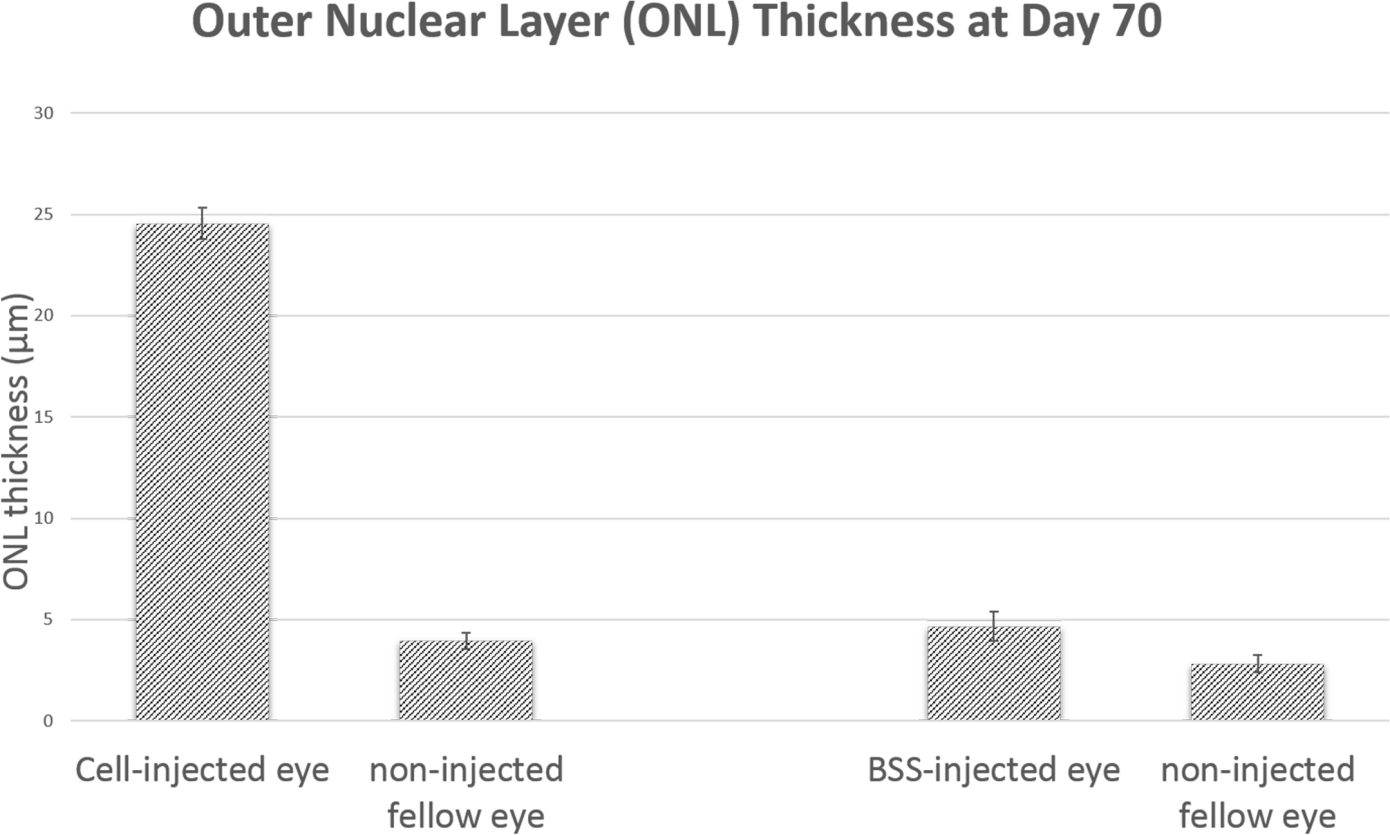
Outer Nuclear Layer Thickness. The average ONL thickness of the hWJ-MSCs and BBS injected group at day 70 post injection. The ONL thickness of the hWJ-MSCs injected eyes was significantly greater than that of the uninjected fellow eyes (p<0.001) and eyes injected with BSS (p<0.001). Error bars indicate standard error of the mean.

A representative histology at day 0 and day 70 of different experimental groups are shown in Fig 1. The ONL (marked with asterisk) was clearly visible in the hWJ-MSCs injected eyes (with approximately 10 cell thickness) but undetectable in the BSS injected eyes nor control eyes. The ONL of the contralateral eye of the hWJ-MSCs injected animals was also undetectable (Fig 1C). The average thickness of the ONL of the hWJ-MSCs injected eyes (24.55 ± 0.79 μm) was significantly greater than that of the uninjected contralateral fellow eyes (3.94 ± 0.42 μm, p<0.001) and eyes injected with BSS (4.65 ± 0.73 μm, p<0.001) (Fig 2).

**Fig 3:**
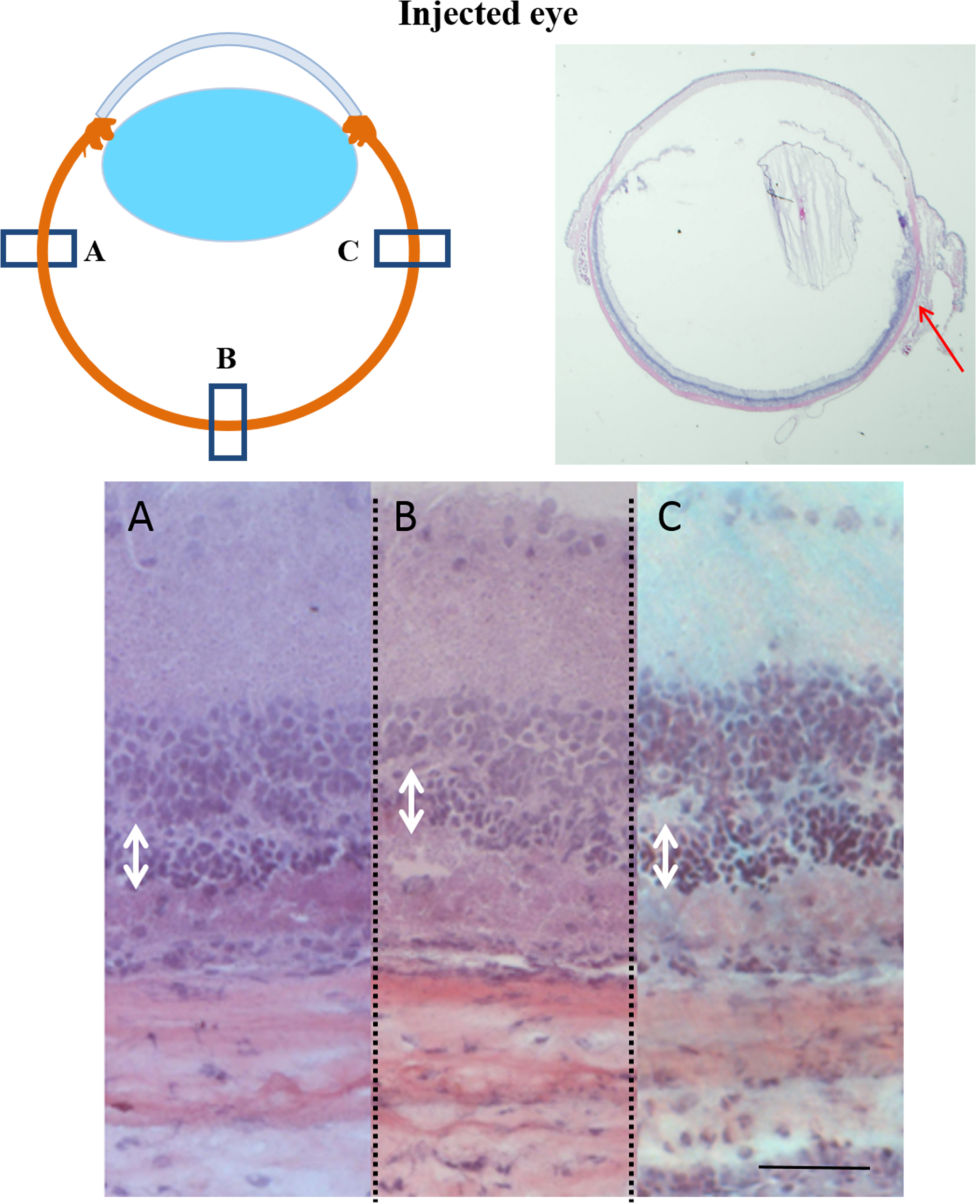
Retinal histology of the injection and non-injection sites at day 70. In eye injected with hWJ-MSCs, there was a generalised preservation of outer nuclear layer (double-headed arrows) over the whole retina as shown at the site of injection (A), middle region (B) and at the site furthest away from the site of injection (C) at day 70. The site of injection was evident by a small remnant subretinal bump (red arrow). This showed that the preservation of outer nuclear layer is not limited to the injection site. Scale bar represents 50 μm.

In the hWJ-MSCs injected eyes, there was a generalised preservation of the outer nuclear layer over the entire retina. The ONL at the site of injection (Fig 3A), middle region (Fig 3B) and at the site furthest away from the site of injection (Fig 3C) are shown in Fig 3. The site of injection was evident by a small remnant subretinal bump. The histology analysis did not show any tumor formation or destruction of laminar structure after cell injection.

The engraftment and survival of hWJ-MSCs were determined by staining the sectioned retinas of the RCS rats with Stem121 at day 70. Stem 121 is a pan human-specific antibody that recognizes only human cytoplasmic protein marker. At day 70, hWJ-MSCs appeared to have survived and migrated progressively to form a continuous layer of cells in the subretinal space around the injected area (staining positive for stem121).

**Fig 4:**
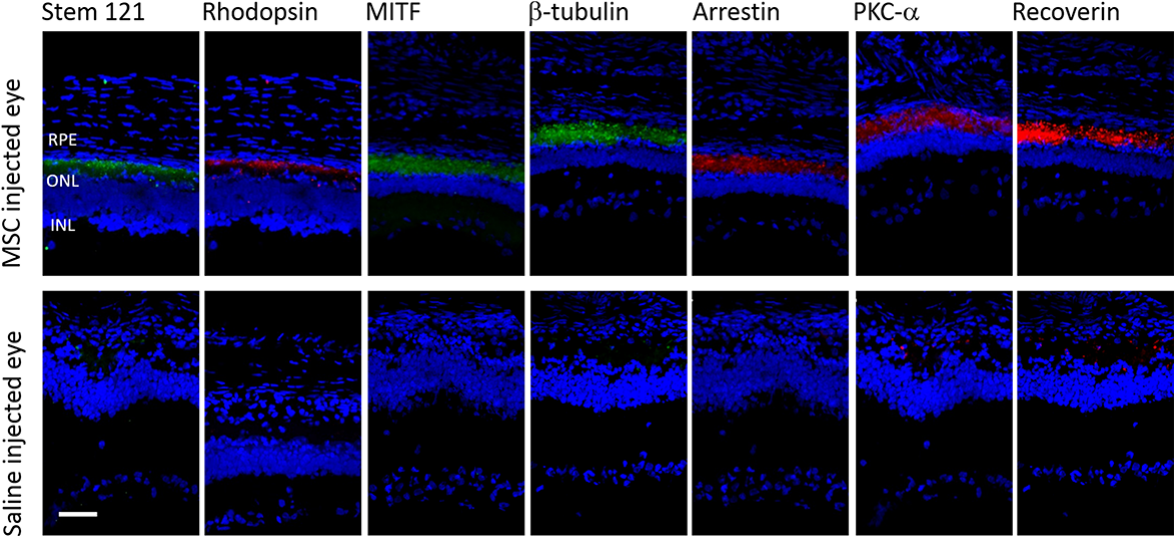
Immunohistochemistry of the injected eyes and control at day 70. Confocal microscopy at day 70 of the hWJ-MSCs and saline injected eye. Positive staining of the retinal antibody markers was detected in the hWJ-MSCs injected eyes but not in the saline injected eye. The antibodies used were Stem 121(green) for mesenchymal stem cells, Rhodopsin (red) for rod photoreceptors, MITF (green) for RPE specific markers, β tubulin (green) for immature neurons, anti-cone arrestin (red) for cone photoreceptors, PKC-α (red) for bipolar cells and recoverin (red) for cone bipolar. All slides were counterstained with DAPI (blue) to label the nucleus. Scale bar (white) represents 1000 μm.

**Fig 5:**
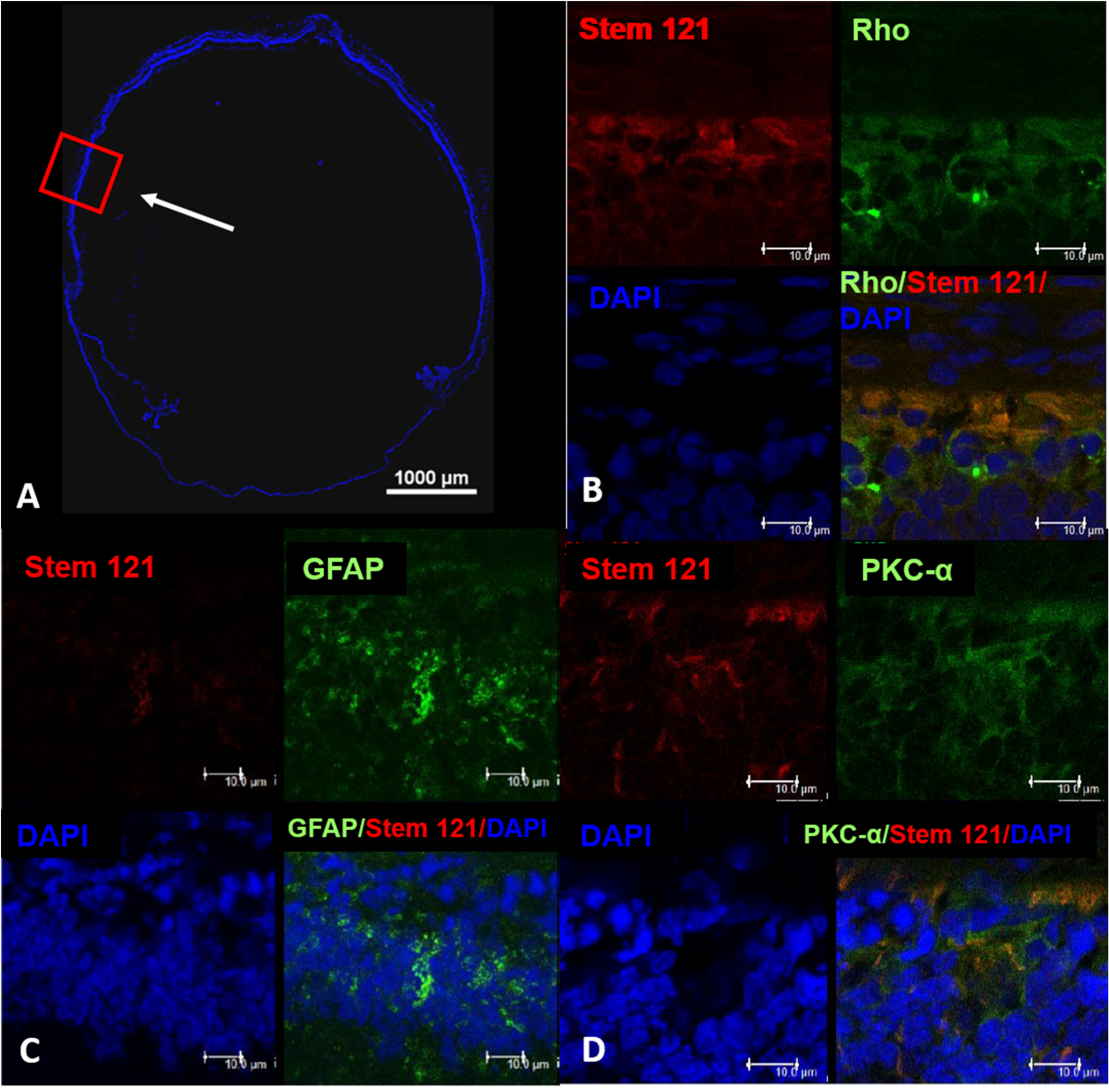
Confocal microscopy of the whole eye injected with hWJ-MSCs. Confocal microscopy of the whole eye (A) and magnified images of the injected site (B-D). Red box represents the magnified area and the white arrow indicates the injection site. Co-localisation of DAPI (blue) and stem 121 (red) with rhodopsin (green), GFAP (green) and PKC-α (green) was detected at day 70 post injection, indicating that hWJ-MSCs has the potential to differentiate to retinal neuronal cells. Scale bar represents 10 μm.

The retina sections expressed both stem 121 and various antibody markers namely rod rhodopsin, protein kinase (PKC-α), glial fibrillary acidic protein-GFAP, cone arrestin, recoverin, microphthalmia-inducing transcription factor (MITF) and ß-tubulin III. These antibody markers stained for both the rat and human cells. At day 70, positive staining of the antibody markers (Fig 4) was detected in eyes injected with hWJ-MSCs but not in the control eyes, suggesting that the injected hWJ-MSCs started to show characteristics of the various retinal neural cells. There was also co-localizations of Stem 121 and photoreceptor markers (Fig 5) indicating that hWJ-MSCs has the potential to differentiate into retinal neural cells.

### Retinal Function

**Fig 6:**
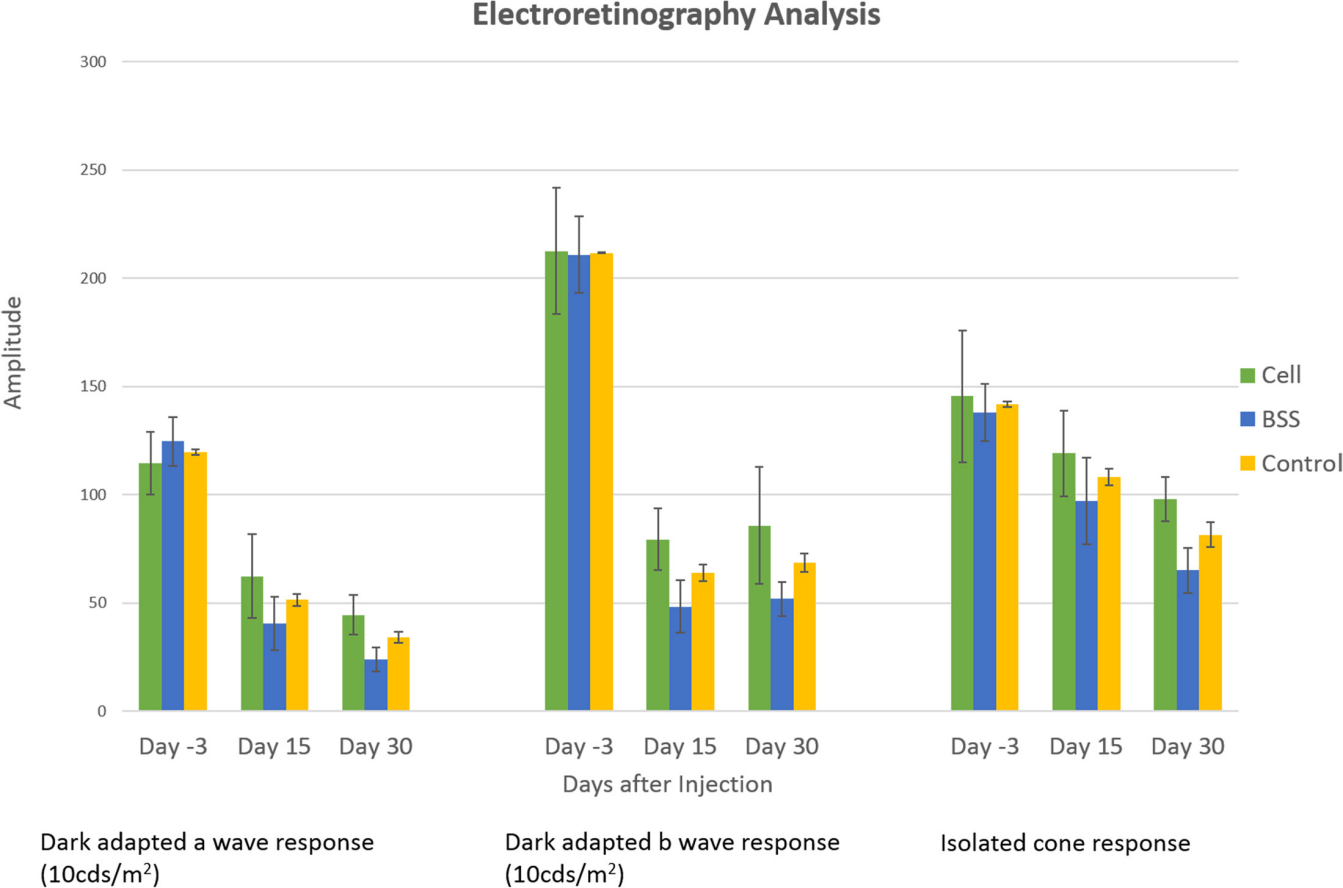
Retinal function. The dark adapted ERG a- and b-wave response amplitudes (10 cd.s/m^2^) and the isolated cone response at days -3, 15 and 30 for each experimental group. Although there was a trend that the ERG amplitude of the injected group was higher than that of the non-injected and control groups at days 15 and 30, the differences in ERG responses between the studied groups were not statistically significant at any time point post injection (p>0.05). All groups showed undetectable ERG at days 70. Error bars indicate standard error of the mean.

The dark adapted ERG a- and b-wave response amplitudes at 10 cd.s/m^2^ and the isolated cone responses at days -3, 15 and 30 for each experimental group are shown in Fig 6. Although there was a trend that the ERG amplitude of the injected group was greater than that of the non-injected and control groups at days 15 and 30, the differences in ERG responses between the studied groups were not statistically significant at any time point post injection. All groups showed undetectable ERG at day 70.

### hWJ-MSCs Migration

**Fig 7:**
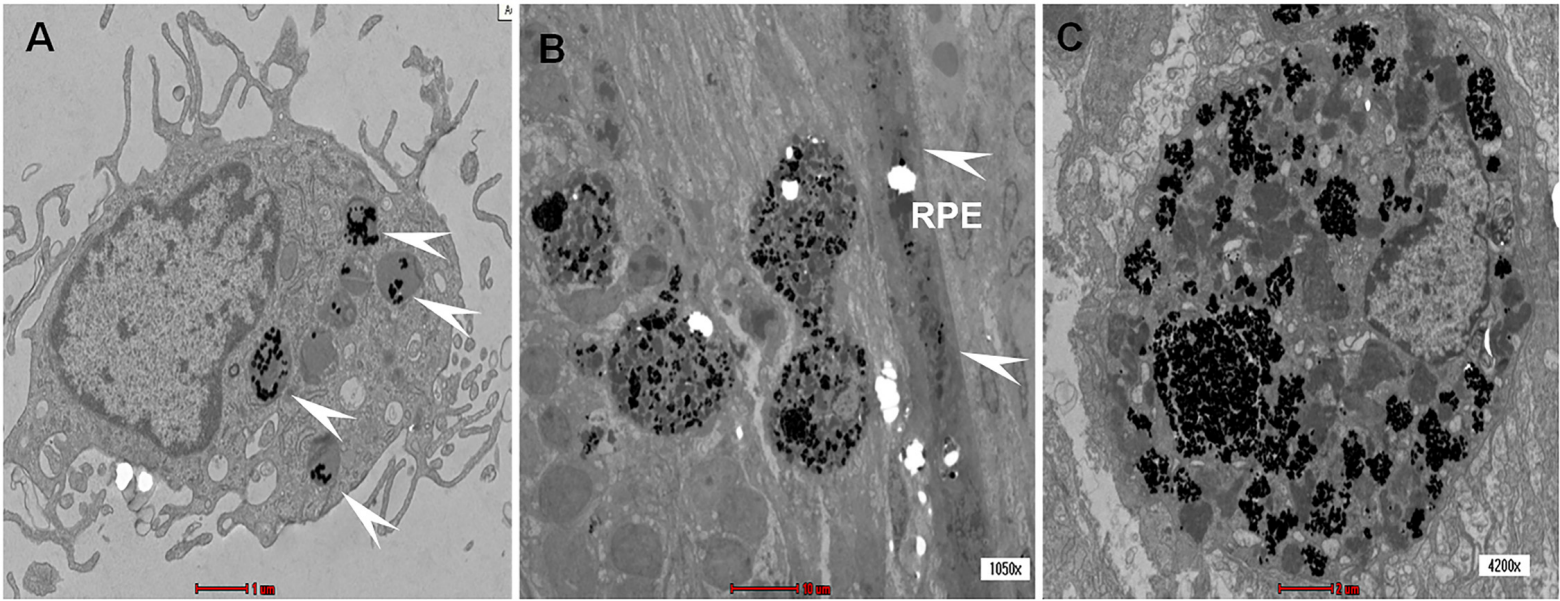
Transmission electron microscopy. Transmission electron microscopy demonstrated the uptake of gold nanoparticles (arrow heads) by the hWJ-MSCs in vitro (A). At day 5 after injection into the cell, the gold nanoparticles laden cell was located in the subretinal space (B) and some of the gold nanoparticles were seen taken up by the retinal pigment (arrow heads), (C) showed a magnified version of the gold laden cell *in vivo*.

**Fig 8:**
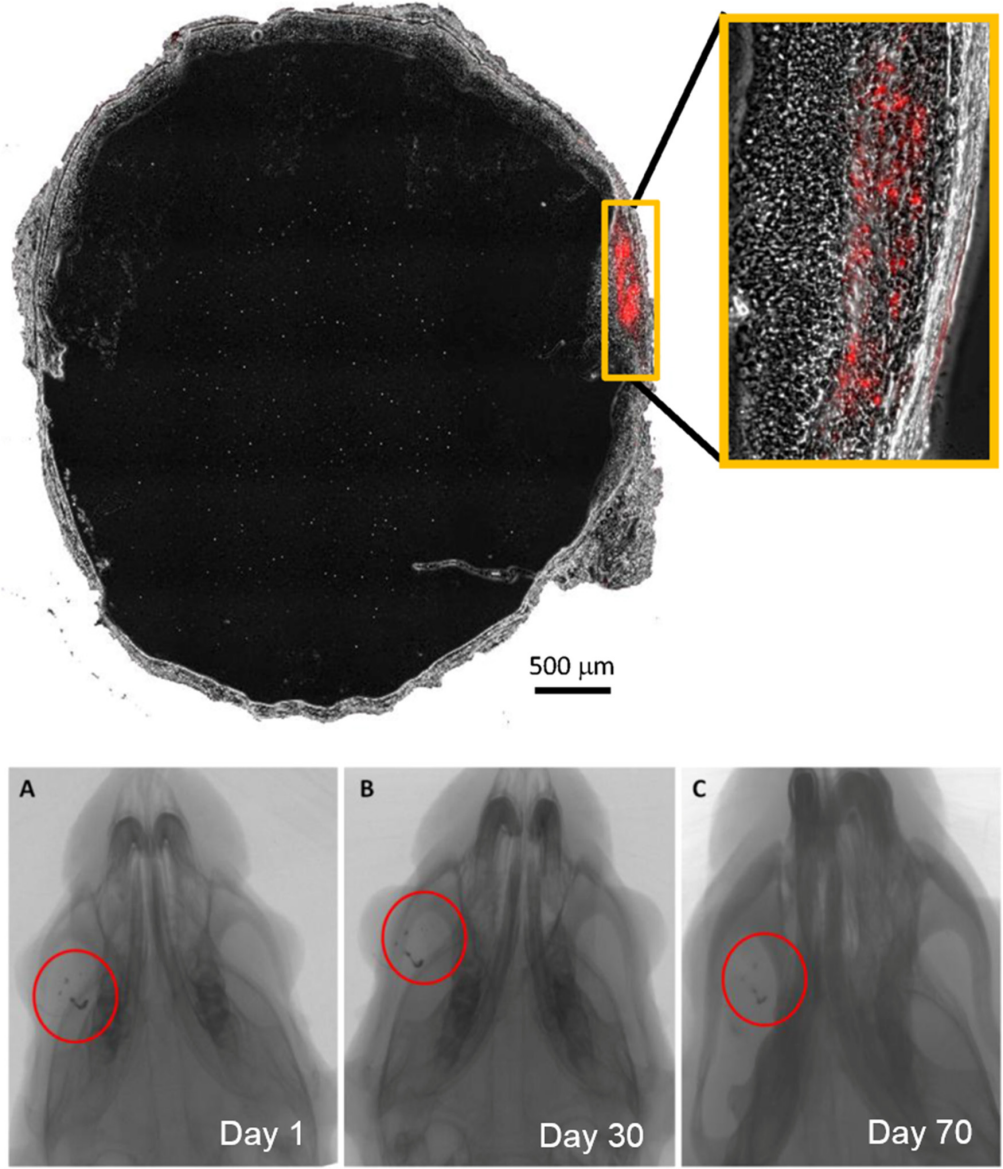
Tracking with micro-computed tomography. MicroCT images showing localisation of gold-loaded hWJ-MSCs in the right eye (A) at day 1 and it remained in the eye with no further migration systemically at day 30 (B) and day 70 (C) post injection. PKH 26 (labelled red) showed the subretinal location of hWJ-MSCs after the injection at week 2.

The subretinal location of hWJ-MSCs was verified with injection of gold-loaded hWJ-MSCs and localised using transmission electron microscopy (TEM) 5 days after the subretinal injection. TEM showed the subretinal localisation of gold-loaded hWJ-MSCs. Some of the gold nanoparticles were seen being phagocytosed by the retinal pigment epithelium layer and retained there (Fig 7). The hWJ-MSCs were also labelled with PKH 26 to determine the location after the injection and confocal microscopy at 2 weeks showed the subretinal location of the injected cells (Fig 8).

Non-invasive longitudinal study were then done in these rats with micro-computed tomography scanning without sacrificing them for better understanding of the biodistribution of hWJ-MSCs after engraftment in rat. Micro-computed tomography was done at day 1,day 30 and day 70 and showed localisation of the gold-loaded hWJ-MSCs to the engrafted eye (Fig 8A, 8B and 8C) (S1 Video, S2 Video and S3 Video) and no migration systemically were seen in whole body scanning. This helps to establish the safety profile of the cell therapy as localised effect is wanted and not for it to migrate systemically.

### Gold Nanoparticles

**Fig 9:**
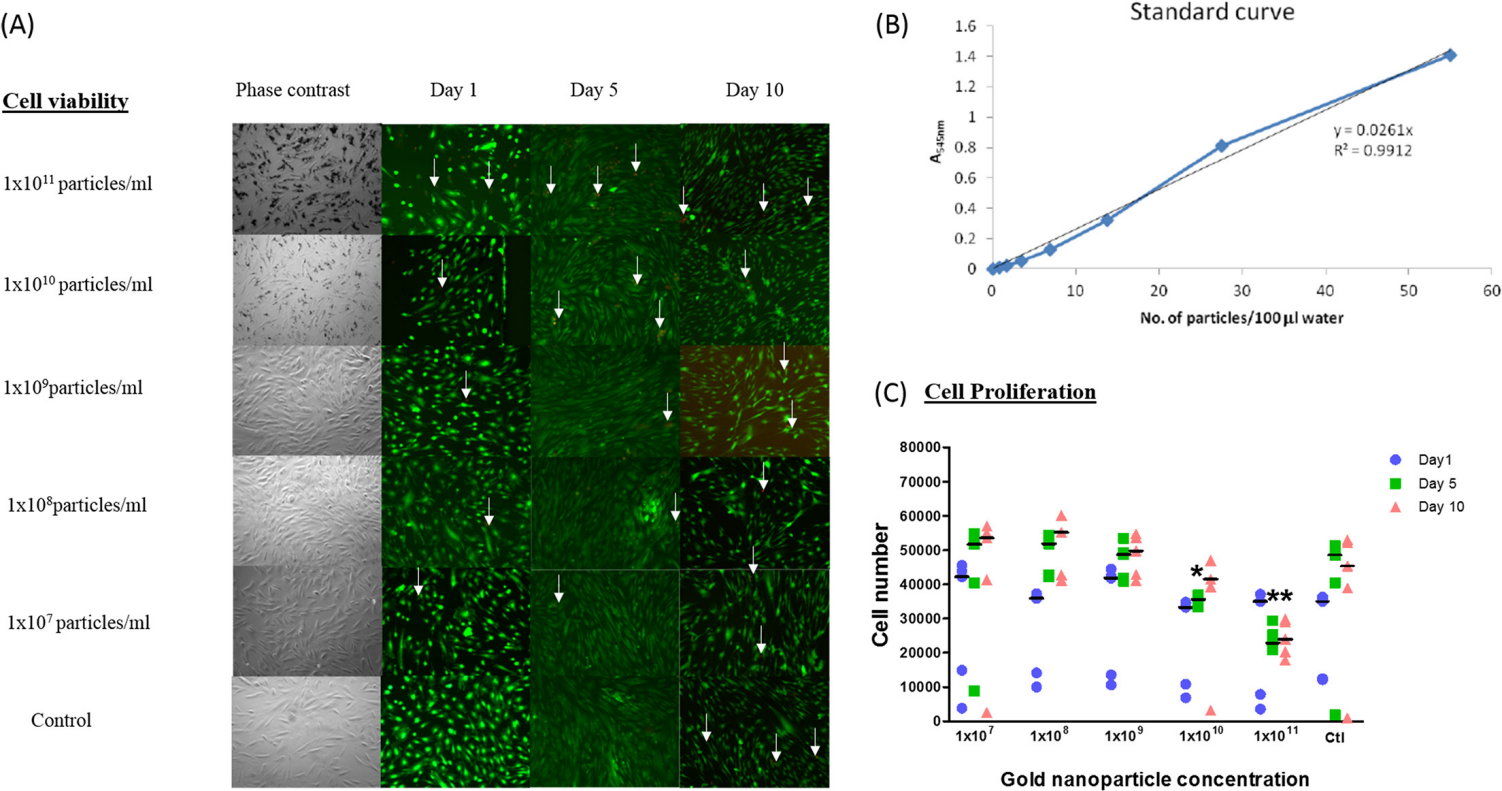
Viability and cytotoxicity of hWJ-MSCs after gold nanoparticles loading. A) Representative LIVE/DEAD images of nanogold-loaded hWJ-MSCs taken at day 1, day 5 and day 10 at different concentration showed good viability where most cells were live cells which were labelled green compared to the very scant dead cells which were labelled red (indicated by white arrow). Scale bar 100μm. B) At optical density with the wavelength of 545 nm, different concentrations of 80 nm gold nanoparticles dissolved in water showed a regression (R^2^) of 0.99. C) The proliferation profile of hWJ-MSCs with various concentration of gold nanoparticles were evaluated over a 10-day period. Median values were represented by horizontal lines. Cell numbers on day 1, day 5 and day 10 are marked with blue circle, green square and pink triangle respectively. At day 1, there was no significant difference in cell count between control sample and samples from any of the gold nanoparticle concentrations. At day 5, the cell count was significantly reduced in the samples incubated with gold nanoparticle concentration of 1x10^10^ particles/ml or higher compared to the control sample (marked with asterisks). The cell count of the samples incubated with gold nanoparticles was also significantly reduced at day 10 compared to the control, but only at the concentration of 1x10^11^ particles/ml (marked with asterisks). (p = 0.014; Mann-Whitney U test).

Nanoparticles of size 80 nm were used in this study. When dissolved in water, the different concentrations of gold in water showed peak absorbance at 545 nm in UV-Vis spectra which is the same as bare gold when dissolved in citrate solution (S5 Fig). The results showed that there was no shift in optimal absorption wavelength for gold nanoparticles in different solutions. At optimal absorption wavelength of 545 nm, the different concentrations of gold nanoparticles showed a regression (R^2^) of 0.99 (Fig 9B). This showed the absence of gold nanoparticles in the supernatant indicating that the exocytosed nanoparticles could be taken up again by the cell or by its neighbouring cells.

After loading hWJ-MSCs with various concentration of gold nanoparticles, viability remained good for all gold nanoparticles concentrations over a 10-day period with minimal cell death. Fig 9A showed that most cells were live cells (labelled green) compared to the very scant dead cells (labelled red marked by white arrows) in the LIVE/DEAD assay.

The proliferation profile of hWJ-MSCs with various concentration of gold nanoparticles are shown in Fig 9C At day 1, there was no significant difference in cell count between control sample and samples from any of the gold nanoparticle concentrations. At day 5, the cell count was significantly reduced in the samples incubated with gold nanoparticle concentration of 1x10^10^ particles/ml or higher compared to the control sample. The cell count of the samples incubated with gold nanoparticles was also significantly reduced at day 10 compared to the control, but only at the concentration of 1x10^11^ particles/ml (p = 0.014). Thus, gold nanoparticles concentration of 1x10^9^ particles /ml was used in the *in vivo* experiment of this study.

## Discussion

In this study, we demonstrate the safety and efficacy of subretinal injection of hWJ-MSCs in delaying retinal degeneration in RCS rats. With regard to the safety, the hWJ-MSCs was localised mainly within the eye, and there was no evidence of systemic distribution as demonstrated by the microCT. There was also no tumorigenesis detected on histology study. In relation to the efficacy, there were a significant preservation of outer nuclear layer in rats injected with hWJ-MSCs compared to control at day 70. However, no significant improvement in retinal function was observed in the treated group. The transplanted hWJ-MSCs expressed protein markers for a number of antibodies, representing different components in the retina at day 70.

The underlying mechanisms of actions are possibly multifactorial. The co-expression of both human STEM 121 and retinal cell markers in this study suggests that the hWJ-MSCs have potential to differentiate into retinal-like cells *in vivo*. This is supported by the successful *in-vitro* induction of hWJ-MSCs in this study to cells exhibiting neuronal markers. Apart from that, other studies had also reported successful *in vitro* induction of hWJ-MSCs into neurons and glia[18] and also retinal progenitor cells[21] indicating the potential of hWJ-MSCs in differentiating into different cell lineages including cells that constitute the retinal layer.

However, the stimulus for *in vivo* differentiation in is still unclear. The diseased microenvironment in which hWJ-MSCs were in or the secretion of neurotrophic factors by hWJ-MSCs creating an inducive microenvironment could have induced cellular differentiation. It is still unknown at the moment and further studies are needed.

Apart from retinal differentiation, another hypothesis towards cellular rescue is the neuroprotective effects by secretion of trophic factors by hWJ-MSCs. These cells had been shown to secrete a number of diffusible neurotrophic factors *in vitro* which include interleukin 6 (IL-6), brain derived neurotrophic factor (BDNF), glial-derived neurotrophic factor (GDNF), neurotrophin 3(NTF3) and fibroblast growth factor (FGF-2)[16,40]. Trophic factors secreted by hWJ-MSCs also showed better neuronal differentiation and neural protection compared with bone marrow derived mesenchymal stem cells [23]. Despite the lack of migration of hWJ-MSCs to cover the entire retina as shown by the micro-computed tomography, it is still able to maintain the preservation of ONL on the opposite side of the retina. This suggests that neuroprotection by the secretion of trophic factors is the most plausible answer. The role of secretome of hWJ-MSCs in retinal regeneration should be studied further in future research and unfortunately, it is one of our limitation of the study at the moment.

The third mechanism of rescue could be by cell fusion where the hWJ-MSCs fuse with the rat cells and adopt the phenotype of the diseased cells, therefore, mediating repair and rescue. Oxidative damage and cell-to-cell contact were postulated to be a prerequisite to stimulate fusion [41]. In our study, the expression of both human and rat markers as shown by the confocal microscopy could be explained by fusion mechanism. The hWJ-MSCs may have fused with the adjacent sick cells and rescue them. Further studies are needed to demonstrate this hypothesis further.

Several factors could contribute to the poor functional results observed in this study. Firstly, the dose of the injection of hWJ-MSCs could be inadequate. An escalating dose could be tested in the future to look for the correct dosage needed for functional efficacy. Multiple injections at different site could also be an option to reach the desired efficacy and achieve a greater area of preservation with multiple sites. Second, it is also possible that the changes in retinal function was minimal and the full-field system is not sensitive enough to detect this changes. A focal or multifocal ERG system would be more sensitive to detect a small or localised functional changes at the injection site.

Another possible explanation is whether the differentiated retinal neuron cells are functional; whether there are synaptic contacts or integration with the remaining retinal cells. The formation of synaptic contacts or integration may take a longer time to establish; more than 70 days post injection of the study period and hence, rescue or restoration of visual function may be seen later. Synaptic markers such as synaptophysin could also be included in future confocal studies. The sample size could also be too small to detect a significant difference.

As for the route of delivery, subretinal route of administration has the advantage of direct implantation to the site of disease where the degenerating photoreceptors and RPE are targeted and therefore yield better results. Intravitreal injection results in clusters intravitreally on the lens posterior surface and also on the retinal surface [42]. The inner limiting membrane of the retina poses a physical barrier preventing the migration of stem cells into the retina. On the other hand, intravenous administration was not favoured due to few limitations. The blood retinal barrier will limit the efficacy and the dose needed to reach therapeutic level may be high apart from the potential effects of systemic exposure to hWJ-MSCs. Data on intravenous stem cell delivery suggest that majority are trapped initially in the lungs[43] and can cause death by pulmonary embolism in small laboratory animals[44]. Intravenous route requires chemoattractant in order for the cells to home onto the site. There are no evidence to suggest that retinitis pigmentosa produces chemoattractant for the cells to reach retina. There was a study showing the potential of MSCs in treating retinal degeneration by intravenous injection but systemic distribution revealed deposition in other tissues including lungs, kidneys and liver [45]. As MSCs have multipotent potentials, another cause of concern is the undesirable effect of enhancing pre-existing tumour or relapses by cell fusion in systemic administration[46,47].

In developing a cell based therapy for retinal degeneration, the safety profile is another concern. Unlike embryonic stem cell which is pluripotent and has unlimited proliferative capacity, hWJ-MSCs has less tendency to form teratoma [48] and in this study, histology showed no tumor formation or abnormal vascularisation after hWJ-MSCs injection. The hWJ-MSCs also stayed localised to the transplanted area in the eye and did not migrate systemically as shown in the micro-computed tomography.

However, another possible explanation for the lack of migration shown by micro-computed tomography could be limitation in the study by decreased gold nanoparticles loading over time due to cell division and other mechanism such as exocytosis; the cell division could cover the entire retina but the signals were too weak to be picked up by micro-computed tomography as they undergone mitosis.

hWJ-MSCs has its attractiveness for a cell based therapy for retinal degeneration. Its advantages include being an ethical cell source, having good proliferative rate, easily available, and has low immunogenicity. Using it off-the-shelf rather than inducing it into differentiated cells would not confine it to its limited capacity but instead allowing it to act through multiple mechanisms as mentioned above with the plasticity to differentiate into different types of retinal neurons rather than confined to a specific type of cell. Long tedious preparation of induction and differentiation may give rise to contamination and errors apart from the burden of high cost consumption. In addition, hereditary conditions such as retinitis pigmentosa, have underlying genetic abnormalities rendering autologous cells inappropriate for such treatment.

From this study, we can see that hWJ-MSCs has the potential as a cell therapy for retinal degeneration. However, this preliminary study has its many limitations and need to be followed up with further study for its many unaddressed issues. In this study, hWJ-MSCs demonstrate the potential to integrate and differentiate into retinal cells; however synapse formation needs to be established for visual function to be restored. Future directions can look into ribbon synapses and function. Secretion of trophic factors *in vivo* and role of cell fusion are other aspects that can be looked into.

In summary, subretinal injection of hWJ-MSCs delays the loss of the photoreceptors in RCS rats. hWJ-MSCs appears to be safe and has potential to differentiate into retinal-like cells. The potential of this cell-based therapy for the treatment of retinal dystrophies warrants further studies.

## Supporting Information

S1 Fig Flow cytometric analysis. Immunophenotyping with flow cytometric analysis showed positive surface markers expression of CD 73, CD90 and CD 105 and negative for hematopoietic markers of CD 34 and CD 45 as well as reduced expression for HLA-DR.
(TIF)

S2 Fig Differentiation ability of hWJ-MSCs into three different lineages. Adipogenic differentiation of hWJ-MSCs with positive staining for oil red O (A), Chondrogenic differentiation with positive staining for Alcian Blue (B) and Osteogenic differentiation with positive staining for Alizarin Red S.
(TIF)

S3 Fig Neuronal differentiation of hWJ-MSCs *in vitro*. Induced hWJ-MSCs showed positive staining for both glial fibrillary acidic protein (GFAP) (A) and nestin (C) which are neuronal markers compared to controls which were non-induced cells (B,D). Scale equals to 100μm.
(TIF)

S4 Fig Injection technique (Figure A). The superotemporal sclera was exposed with traction suture on conjunctiva **(Figure B).** Conjunctival periotomy was performed to expose the bare sclera **(Figure C).** A sclera tunnel track was created with a 30G needle and cell injected with Hamilton syringe. A bleb under the sclera was seen after the injection **(Figure D).** Site of injection marked with a suture.
(TIF)

S5 Fig Optimal absorption wavelength of gold nanoparticles. Line graph showing different concentrations of gold nanoparticles dissolved in water and bare gold in citrate solution exhibited similar peak absorbance wavelength at 545nm in UV-Vis spectra and photo of 80nm citrate-stabilized gold nanoparticles in water.
(TIF)

S1 Table Gene expression for post-thaw hWJ-MSCs relative to GAPDH. (TIF)

S1 Video MicroCT 3D configuration of eye injected with gold-loaded hWJ-MSCs at day 1. (MP4)

S2 Video MicroCT 3D configuration of eye injected with gold-loaded hWJ-MSCs at day 30. (MP4)

S3 Video MicroCT 3D configuration of eye injected with gold-loaded hWJ-MSCs at day 70. 3D configuration with MicroCT showing localization of gold-loaded hWJ-MSCs to the site of injection till day 70.
(MP4)
