# Supplementary figures and images for: Safety and Efficacy of Human Wharton's Jelly-Derived Mesenchymal Stem Cells Therapy for Retinal Degeneration
Source: PLoS One. 2015 Jun 24;10(6):e0128973. doi: 10.1371/journal.pone.0128973 (PMC4479609; doi:10.1371/journal.pone.0128973)

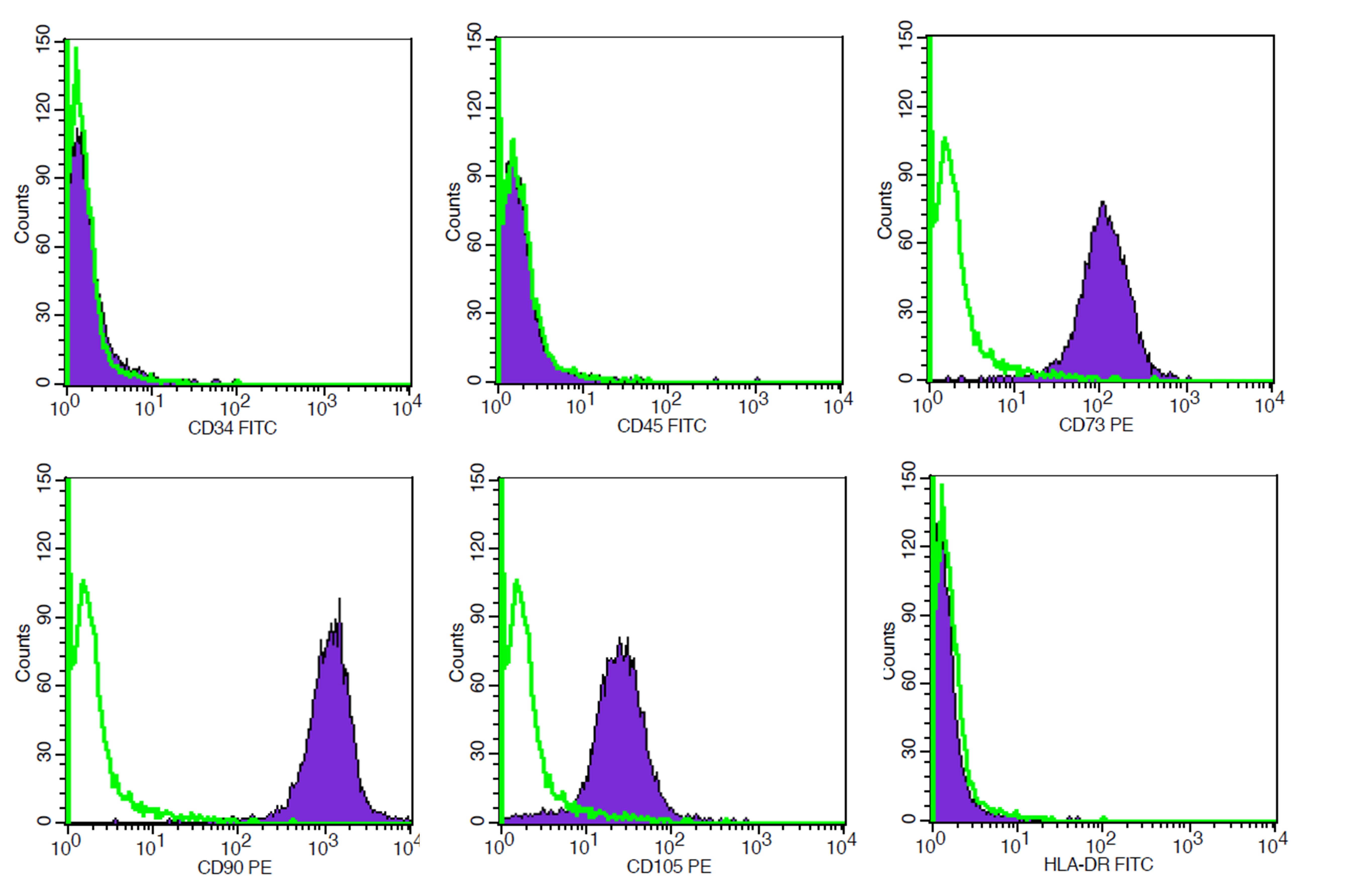

Supplement: S1 Fig — Immunophenotyping with flow cytometric analysis showed positive surface markers expression of CD 73, CD90 and CD 105 and negative for hematopoietic markers of CD 34 and CD 45 as well as reduced expression for HLA-DR. (TIF) [file pone.0128973.s001.tif]

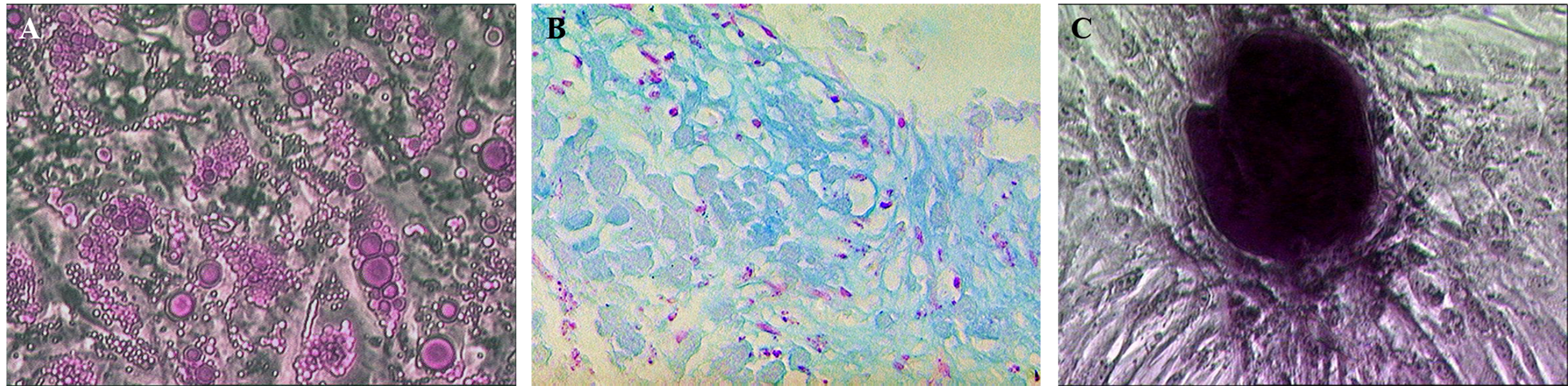

Supplement: S2 Fig — Adipogenic differentiation of hWJ-MSCs with positive staining for oil red O (A), Chondrogenic differentiation with positive staining for Alcian Blue (B) and Osteogenic differentiation with positive staining for Alizarin Red S. (TIF) [file pone.0128973.s002.tif]

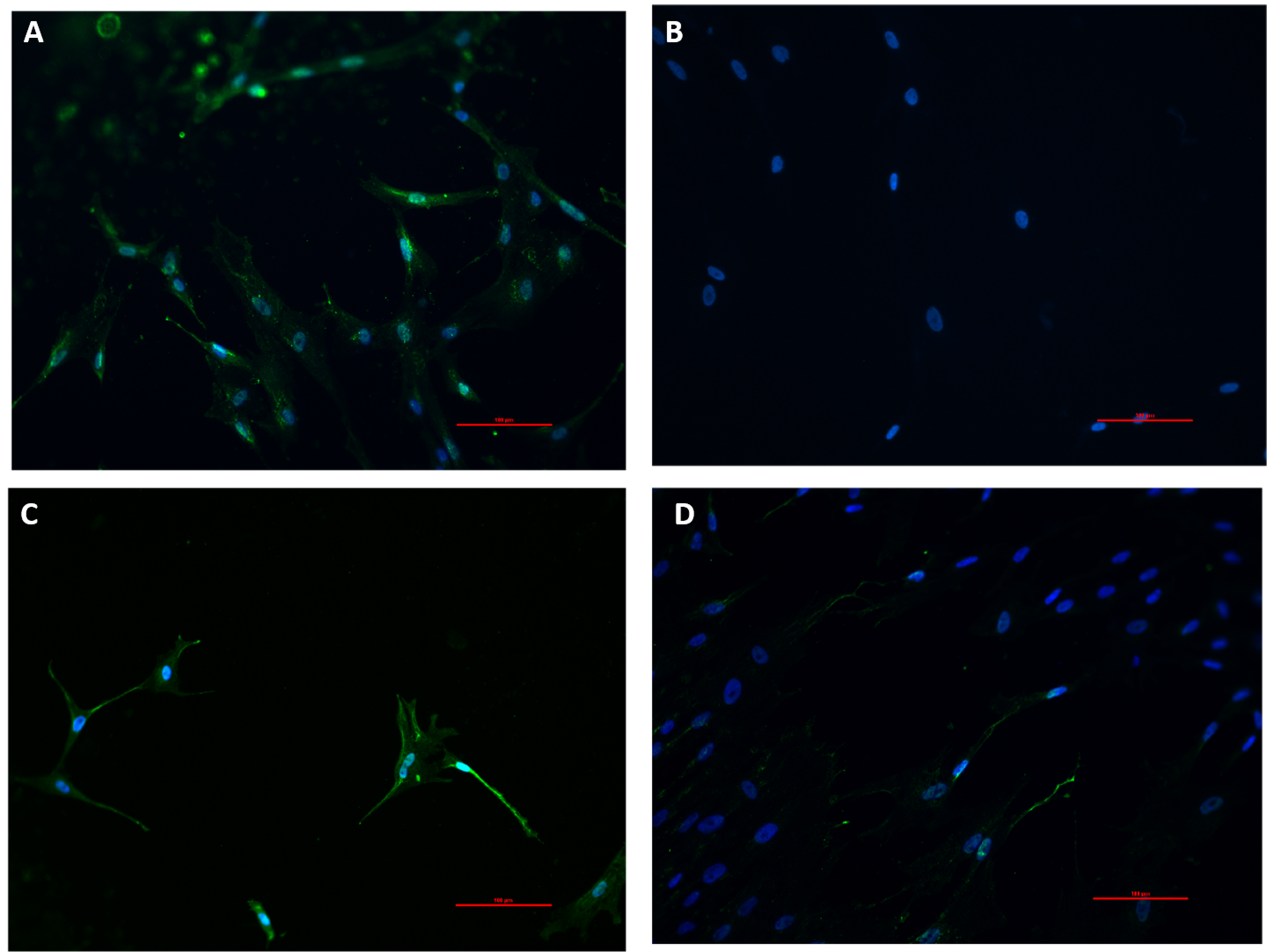

Supplement: S3 Fig — Induced hWJ-MSCs showed positive staining for both glial fibrillary acidic protein (GFAP) (A) and nestin (C) which are neuronal markers compared to controls which were non-induced cells (B,D). Scale equals to 100μm. (TIF) [file pone.0128973.s003.tif]

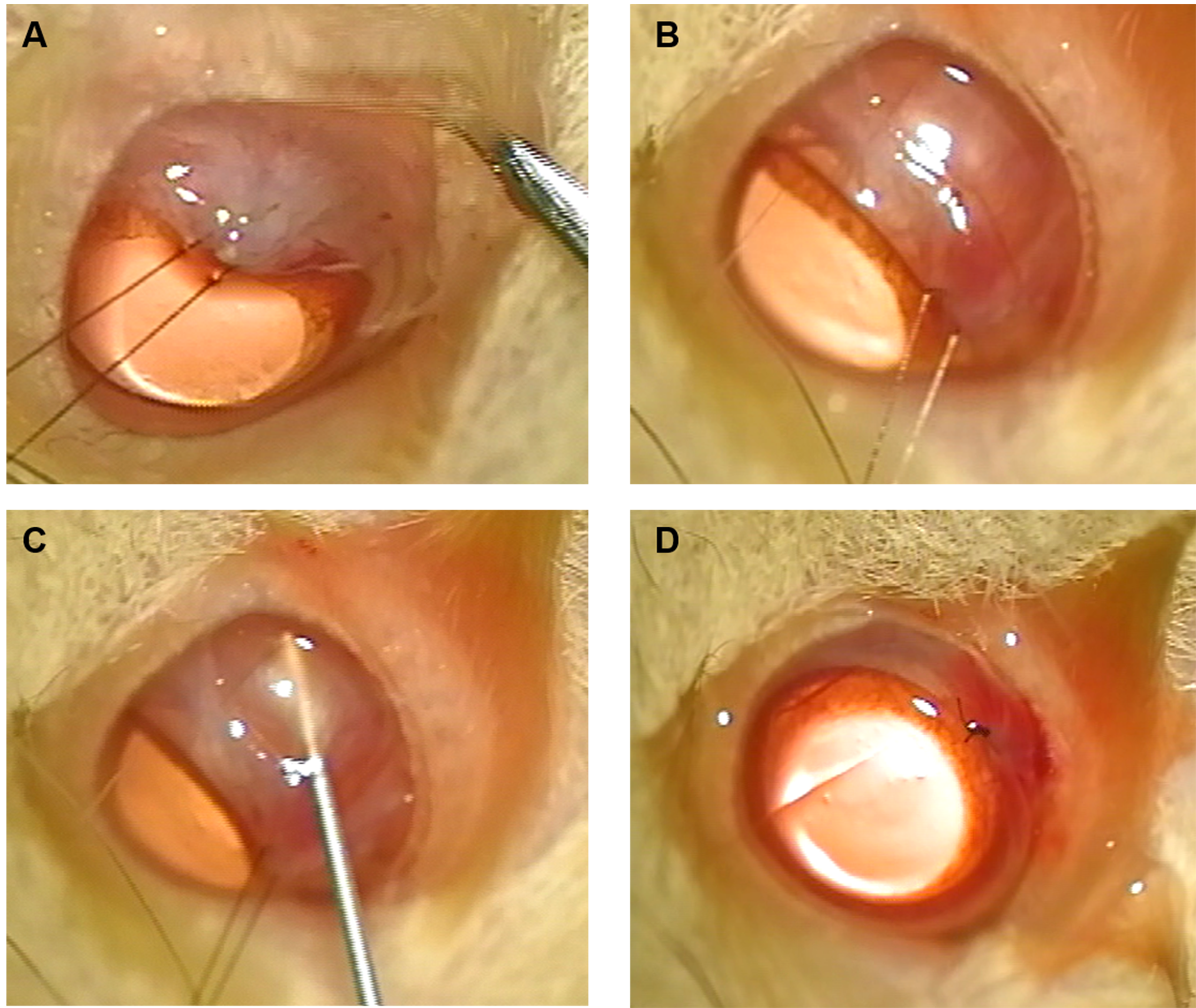

Supplement: S4 Fig — The superotemporal sclera was exposed with traction suture on conjunctiva (Figure B). Conjunctival periotomy was performed to expose the bare sclera (Figure C). A sclera tunnel track was created with a 30G needle and cell injected with Hamilton syringe. A bleb under the sclera was seen after the injection (Figure D). Site of injection marked with a suture. (TIF) [file pone.0128973.s004.tif]

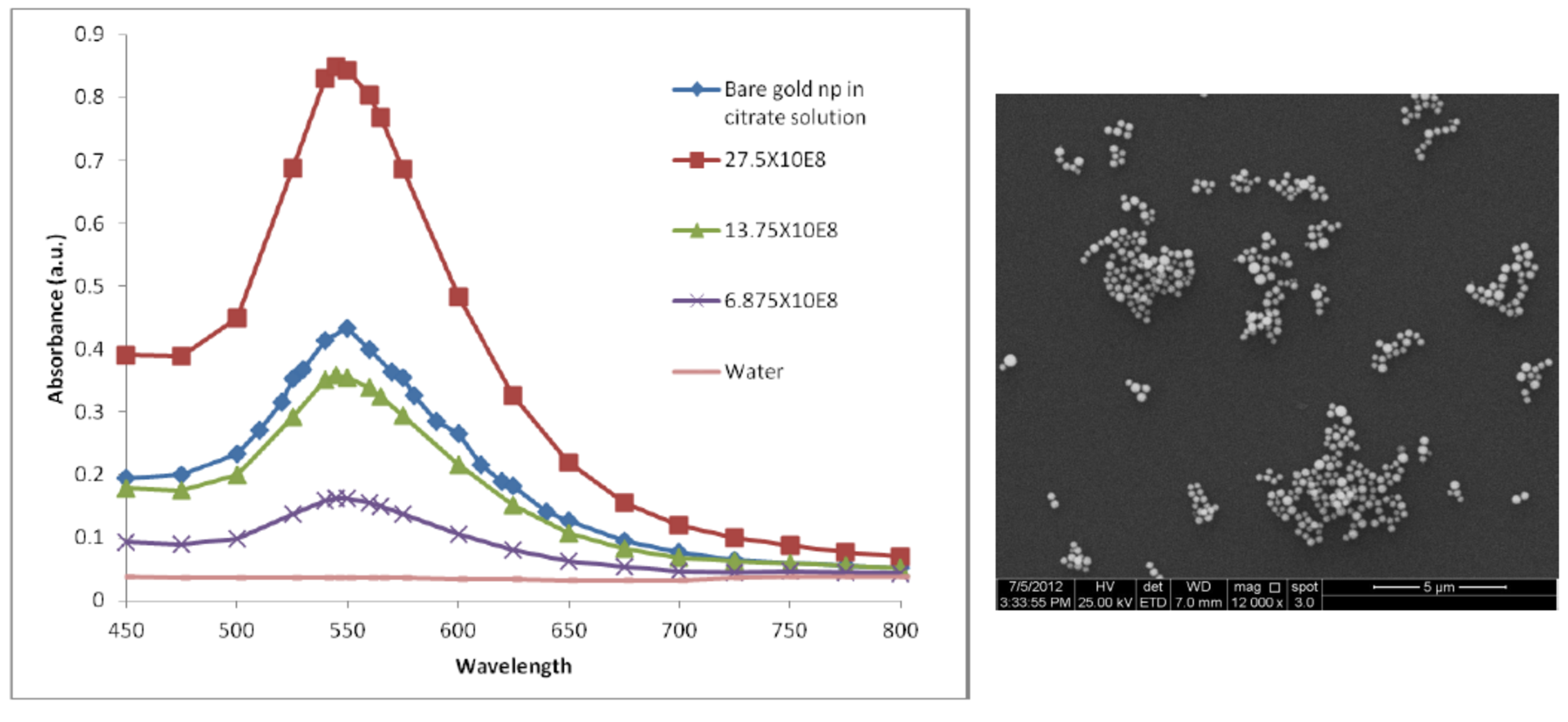

Supplement: S5 Fig — Line graph showing different concentrations of gold nanoparticles dissolved in water and bare gold in citrate solution exhibited similar peak absorbance wavelength at 545nm in UV-Vis spectra and photo of 80nm citrate-stabilized gold nanoparticles in water. (TIF) [file pone.0128973.s005.tif]

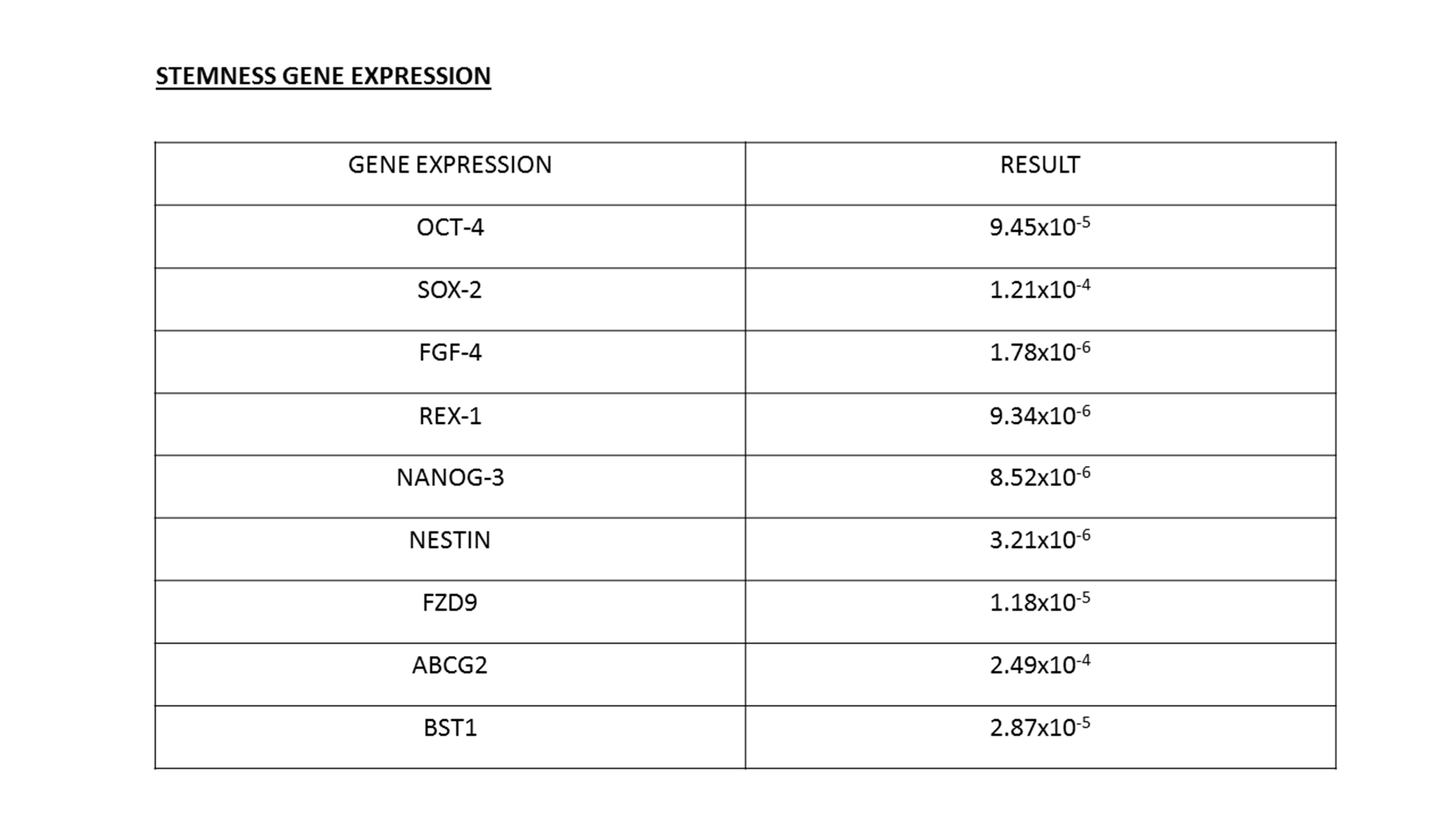

Supplement: S1 Table — (TIF) [file pone.0128973.s006.tif]
